# Prediction of hot spots in protein–DNA binding interfaces based on supervised isometric feature mapping and extreme gradient boosting

**DOI:** 10.1186/s12859-020-03683-3

**Published:** 2020-09-17

**Authors:** Ke Li, Sijia Zhang, Di Yan, Yannan Bin, Junfeng Xia

**Affiliations:** 1 School of Information and Computer, Anhui Agricultural University, Hefei, 230036 Anhui China; 2 Key Laboratory of Intelligent Computing and Signal Processing of Ministry of Education, Institutes of Physical Science and Information Technology, Anhui University, Hefei, 230601 Anhui China; 3 School of Life Sciences, Anhui University, Hefei, 230601 Anhui China

**Keywords:** Protein–DNA complexes, Hot spot, Supervised isometric feature mapping, Extreme gradient boosting

## Abstract

**Background:**

Identification of hot spots in protein-DNA interfaces provides crucial information for the research on protein-DNA interaction and drug design. As experimental methods for determining hot spots are time-consuming, labor-intensive and expensive, there is a need for developing reliable computational method to predict hot spots on a large scale.

**Results:**

Here, we proposed a new method named sxPDH based on supervised isometric feature mapping (S-ISOMAP) and extreme gradient boosting (XGBoost) to predict hot spots in protein-DNA complexes. We obtained 114 features from a combination of the protein sequence, structure, network and solvent accessible information, and systematically assessed various feature selection methods and feature dimensionality reduction methods based on manifold learning. The results show that the S-ISOMAP method is superior to other feature selection or manifold learning methods. XGBoost was then used to develop hot spots prediction model sxPDH based on the three dimensionality-reduced features obtained from S-ISOMAP.

**Conclusion:**

Our method sxPDH boosts prediction performance using S-ISOMAP and XGBoost. The AUC of the model is 0.773, and the F1 score is 0.713. Experimental results on benchmark dataset indicate that sxPDH can achieve generally better performance in predicting hot spots compared to the state-of-the-art methods.

## Background

Protein-DNA interactions play a crucial role in many biological processes, such as gene transcription and translation, DNA repair and assembly [1, 2]. In pioneering research work on the binding of human growth hormone to its receptor, a small number of interface residues, known as hot spots, were found to contribute more affinity compared with other amino acid residues [3]. In the experiments, alanine scanning mutation technology has been used to identify hot spots when their free energy changes exceed a certain threshold [4]. This experimental method was also used to explore the mechanism of protein-DNA recognition. As the experimental method is high-cost and time-consuming, the computational method provides another way for studying hot spots prediction.

A series of methods based on feature selection have been used to study the hot spots in protein binding interfaces. Xia et al. selected the three optimal features with the largest contribution through a two-step feature selection approach including maximum relevance minimum redundancy (mRMR) and exhaustive search [5]. Pan et al. used gradient tree boosting algorithm to find the smallest optimal features from 125 candidates [6]. Qiao et al. proposed a hybrid feature selection strategy, combining the feature subsets selected by decision tree and mRMR respectively, and finally obtained six features using pseudo sequential forward selection [7]. Deng et al. adopted a two-step feature selection method consisting of mRMR and sequential forward selection (SFS) to select the best 6 features from a group of 156 features [8]. Hot spots identification is of great significance for exploring the potential binding mechanism and the stability of protein-DNA interactions [9]. So far, many studies have focused on the prediction of binding sites in protein-DNA complexes [10]. However, there is little research on the prediction of hot spots in protein-DNA complexes. Recently, Zhang et al. used a computational approach to predict the hot spots in protein-DNA binding interfaces [11].

The above methods have some disadvantages. For example, the mRMR-based method has good time performance, but its classification accuracy is general and it cannot eliminate redundancy completely [12]. Although the SFS-based method has good feature resolution, it has high computational complexity and is easy to over-fit [13]. Manifold learning is a nonlinear dimensionality reduction method appeared in recent years. It can map the high-dimensional input data to the low-dimensional manifold and preserve the topological structure of the data while reducing the dimension. The classical manifold learning methods include isometric feature mapping (ISOMAP) [14], local linear embedding (LLE) [15], etc. However, these are unsupervised dimensionality reduction methods, which cannot make full use of the class label information of samples. Here, we propose a new method based on supervised manifold learning to predict the hot spots in protein-DNA binding interfaces. We extracted 64 DNA-binding proteins and collected 114 features based on our previous work [11]. In order to improve prediction performance, supervised isometric feature mapping (S-ISOMAP) [16] algorithm considering the class label information was used to implement dimensionality reduction. Finally, we employed an improved version of the Gradient Boosting algorithm, extreme gradient boosting (XGBoost) [17], to build the prediction model. Experimental results show that compared with the state-of-the-art prediction methods, our method sxPDH (S-ISOMAP and XGBoost based model for prediction of protein-DNA binding hot spots) has higher prediction performance.

## Methods

### Dataset and features used in this work

In this study, we used the same dataset and features as our previous work [11]. Among 64 protein-DNA complexes, 40 complexes were selected randomly as the training dataset including 62 hot spots and 88 non-hot spots and the other 24 complexes were used as the test dataset with 26 hot spots and 38 non-hot spots. We obtained 114 features from four feature groups, namely, solvent accessible surface area, sequence, structure and network. For details, the interested readers can refer to our previous work [11].

### Feature dimensionality reduction

If the dimension of the features is too high, the classifier will over-fit. Therefore, in order to improve the prediction performance of classifiers, reducing the feature dimension is essential. Here, we used S-ISOMAP algorithm, which can make the data of the same category close to and different categories distant from each other in the dimension reduction space, thus achieve dimensionality reduction. The framework of manifold learning algorithm based on S-ISOMAP is as follows [16].

Step 1: Define the dissimilarity distance:

Assuming that the given data are (*x*_*i*_, *y*_*i*_), where *x*_*i*_ ∈ *R*^*D*^(*i* = 1, 2, …, *N*), *y*_*i*_ is the category label for *x*_*i*_, we define the dissimilarity between two points *x*_*i*_ and *x*_*j*_ as [16]:

1 $$ D\left({x}_i,{x}_j\right)=\left\{\begin{array}{c}\sqrt{1-\exp \left(-{d}^2\left({x}_i,{x}_j\right)/\beta \right)}{y}_i={y}_i\\ {}\sqrt{\exp \left({d}^2\left({x}_i,{x}_j\right)/\beta \right)}-\alpha {y}_i\ne {y}_i\end{array}\right. $$

where *d*(*x*_*i*_, *x*_*j*_) represents the Euclidean distance between *x*_*i*_ and *x*_*j*_, the parameter *β* is used to control the growth rate of *D*(*x*_*i*_, *x*_*j*_), and the parameter *α* is used to control the distance between different classes [16].

Step 2: Construct the neighborhood graph:

Firstly the dissimilarity distance between the sample point *x*_*i*_ ∈ *R*^*D*^ and sample points *x*_*j*_ ∈ *R*^*D*^ is calculated [16]. When *x*_*j*_ is one of the nearest *K* points of *x*_*i*_, they are adjacent, that is, there is edge *x*_*i*_*x*_*j*_ in the graph *G* (k-neighborhood). If *x*_*j*_ is not the nearest *K* points of *x*_*i*_, and the Euclidean distance between *x*_*i*_ and *x*_*j*_ is less than the fixed value *ε*, it is considered that there is edge *x*_*i*_*x*_*j*_ in the graph *G* (*ε*-neighborhood). Here, the weight of the edge is set to dissimilarity distance *D*(*x*_*i*_, *x*_*j*_) [16].

Step 3: Compute the shortest paths:

We initialize the shortest path *d*_*G*_(*x*_*i*_, *x*_*j*_) = *D*(*x*_*i*_, *x*_*j*_), if there’s an edge *x*_*i*_*x*_*j*_ in graph *G*; Otherwise *d*_*G*_(*x*_*i*_, *x*_*j*_) = ∞. Then we calculate *d*_*G*_(*x*_*i*_, *x*_*j*_) for each data (*x*_*i*_, *y*_*i*_) [16]:

2 $$ {d}_G\left({x}_i,{x}_j\right)=\min \left\{{d}_G\left({x}_i,{x}_j\right),{d}_G\left({x}_i,{x}_l\right)+{d}_G\left({x}_l,{x}_j\right)\right\} $$

where *l* = 1, 2, …, *N*.

In this way, the shortest path distance matrix ***D***_*G*_ = {*d*_*G*_(*x*_*i*_, *x*_*j*_)} can be obtained. This process is called Floyd algorithm [16].

Step 4: Construct *d*-dimensional embedding:

Multidimensional scaling (MDS) [18] is applied to the distance matrix ***D***_*G*_. The global low-dimensional coordinates are obtained by minimizing the cost function *E*:

3 $$ E={\left\Vert \tau \left({\boldsymbol{D}}_G\right)-\tau \left({\boldsymbol{D}}_Y\right)\right\Vert}_{L^2} $$

where the operator *τ* is defined by *τ*(***D***) =  − ***HSH***/2, in which *H* = {*H*_*ij*_} = {*δ*_*ij*_ − 1/*N*} is the “centering matrix”, and ***S*** = {*S*_*ij*_} = {*D*^2^(*x*_*i*_, *x*_*j*_)} is the square distance matrix. The eigenvector corresponding to the maximum *d* eigenvalues *λ*_1_, *λ*_2_, ⋯, *λ*_*d*_ of *τ*(***D***_*G*_) is *u*_1_, *u*_2_, ⋯, *u*_*d*_ [16]. Then $Y=\mathit{\operatorname{diag}}\left({\lambda}_1^{1/2},{\lambda}_2^{1/2},\cdots, {\lambda}_d^{1/2}\right){\left[{u}_1,{u}_2,\cdots, {u}_d\right]}^T$ is the *d*-dimensional embedding result [16].

### Model construction

XGBoost has achieved the most advanced results in many machine learning challenges based on the idea of continuously reducing the residual of the previous model in the gradient direction to obtain a new model. As an improved version of the Gradient Boosting algorithm, XGBoost performs a second-order Taylor expansion on the loss function to obtain the optimal solution for the regular term outside the loss function. The advantages of multi-core CPU parallel computing is fully utilized to improve the accuracy and speed. Therefore, we established a prediction model for hot spots in protein-DNA binding interfaces based on XGBoost. In order to achieve good experimental results, the XGBoost was tuned using a grid search method, and obtained the optimal parameters with n_estimators = 500, learning_rate = 0.1, and max_depth = 30.

### Evaluation criteria

The computer model used in the simulation is an ASUS FX503VD, the CPU is a dual-core processor i7-7700HQ model with a main frequency of 2.8 GHz, and its memory is 8G. In order to improve the robustness of the prediction model, we used 10-fold cross validation and performed 20 experiments to obtain average results. To evaluate the classification performance of our model, we adopted some commonly used evaluation metrics, including sensitivity (SEN), specificity (SPE), precision (PRE), F1 score (F1), accuracy (ACC), and Matthews correlation coefficient (MCC) [19–23]:

4 $$ SEN= TP/\left( TP+ FN\right) $$

5 $$ SPE= TN/\left( TN+ FP\right) $$

6 $$ PRE= TP/\left( TP+ FP\right) $$

7 $$ F1=\frac{2\times SEN\times PRE}{SEN+ PRE} $$

8 $$ ACC=\frac{TP+ TN}{TP+ TN+ FP+ FN} $$

9 $$ MCC=\frac{TP\times TN- FP\times FN}{\sqrt{\left( TP+ FP\right)\left( TP+ FN\right)\left( TN+ FP\right)\left( TN+ FN\right)}} $$

where TP, FP, TN, FN represent the number of true positive (correctly predicted hot spot residues), false positive (non-hot spot residues incorrectly predicted as hot spots), true negative (correctly predicted non-hot spot residues) and false negative (hot spot residues incorrectly predicted as non- hot spots), respectively. We also adopted the ROC curve as the assessment criteria in this work. From the ROC curve, we calculated the area under the ROC curve (AUC).

## Results and discussion

### Overview of sxPDH

**Fig. 1:**
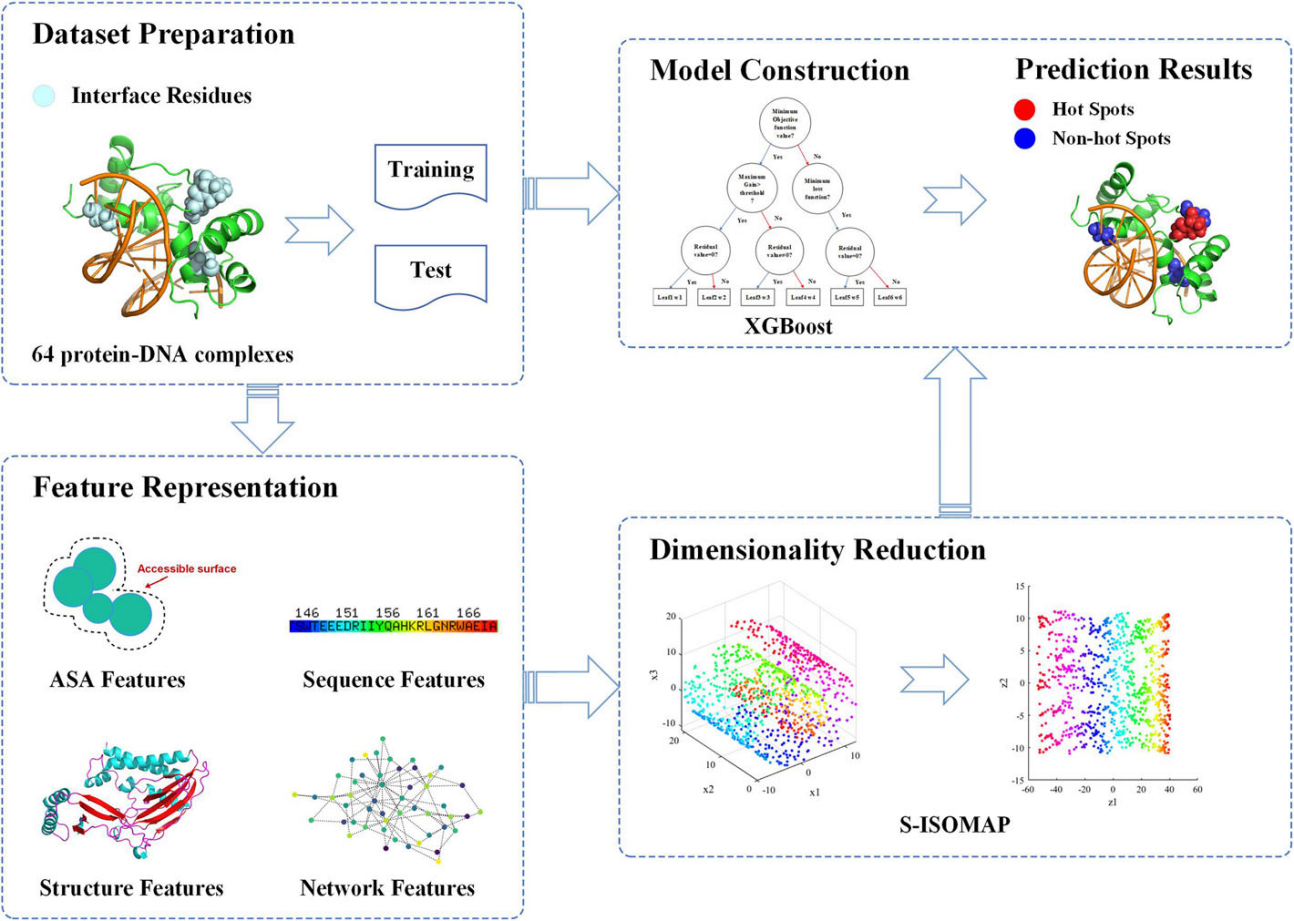
The workflow of sxPDH

Figure 1 shows the workflow of our method sxPDH. First, a benchmark dataset consisting of 88 hot spots and 126 non-hot spots from 64 protein-DNA complexes was constructed. Then, four types of features were generated, namely, solvent-accessible surface area, sequence features, structural features and network features. S-ISOMAP algorithm was then used to reduce the dimension of these feature. On this basis, XGBoost was applied to construct a prediction model of hotspots in protein-DNA binding interface. Finally, according to the feature set after dimensionality reduction, the prediction results are output through the XGBoost model.

### Evaluation of different manifold learning methods

In this study, we reduce feature dimension based on the S-ISOMAP. In order to evaluate the practicability of the S-ISOMAP method, it is compared with three other manifold learning-based methods, including LLE, ISOMAP and supervised locally linear embedding (SLLE) [24], with the XGboost is used as the classification model. LLE method is to obtain low-dimensional embedded coordinates by linear reconstruction of local neighborhood in high-dimensional data, thereby keeping the neighborhood relationship of high-dimensional data unchanged. The goal of ISOMAP method is to maintain the geodesic distance between the points in the original data set to the greatest extent. Both methods are based on unsupervised dimensionality reduction. SLLE introduces class labels by calculating the maximum Euclidean distance between classes, which is based on supervised dimensionality reduction. Table 1 shows the performance of the model using S-ISOMAP compared with the other three manifold learning methods on the test set. From these evaluation criteria, it can be seen that the model prediction effect using S-ISOMAP is the best (PRE = 0.707, F1 = 0.713, MCC = 0.508 and ACC = 0.768).

**Table 1:** Performance of different manifold learning methods on the test set

Method	SEN	SPE	PRE	F1	ACC	MCC	AUC
LLE (10)	0.653	0.711	0.607	0.629	0.687	0.361	0.693
ISOMAP (10)	0.687	0.766	0.692	0.695	0.709	0.476	0.738
SLLE (3)	0.671	0.732	0.648	0.656	0.691	0.381	0.703
S-ISOMAP (3)	**0.707**	**0.819**	**0.721**	**0.713**	**0.768**	**0.508**	**0.773**

The highest value in each column is shown in bold. The numbers in parentheses represent the feature dimensions after dimensionality reduction

**Fig. 2:**
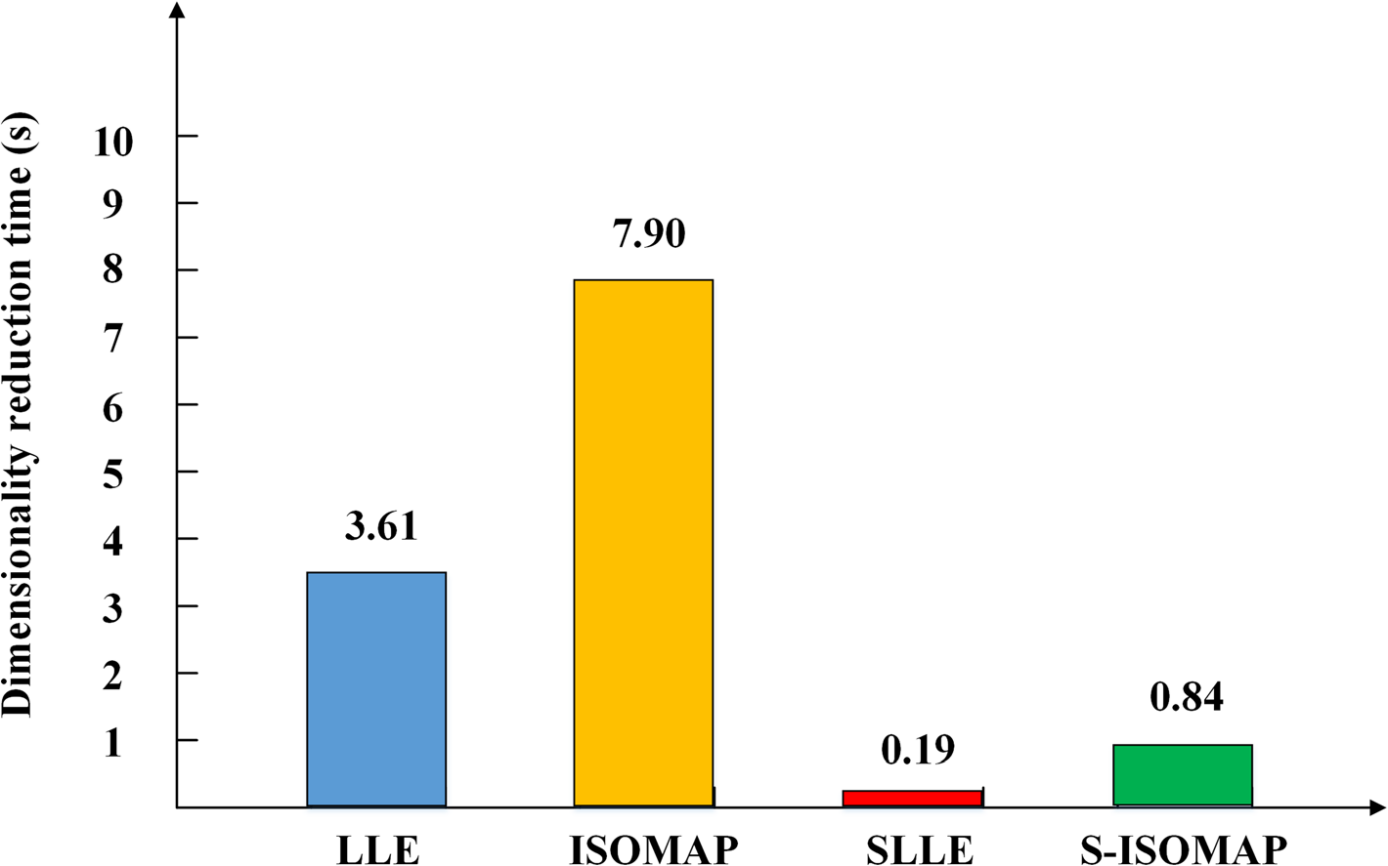
Running time of different manifold learning methods

Figure 2 shows the runtime comparison of our method with the other three manifold learning methods. The dimensionality reduction time of S-ISOMAP is slightly higher than that of SLLE, but lower than those of LLE and ISOMAP.

### Compared with the feature selection methods

To further verify the performance of our model, we also compared its performance with four commonly used feature selection methods with the classification model XGboost. These methods are RF-based on sequential forward selection (RF-SFS) [25], mRMR [26], SVM-based recursive feature elimination (SVM-RFE) [27] and variable selection using random forests (VSURF) [28]. RF-SFS uses RF to rank the importance of features and then performs feature selection using sequential forward selection strategy. The mRMR method analyzes and evaluates features by producing a feature list based on the maximum relevance and minimum redundancy criteria. SVM-RFE is an application of RFE using the weight magnitude as the ranking standard. VSURF adopts a two-stage strategy. It first uses the importance score based on the random forest to sort features, and then uses a stepwise forward strategy to return a smaller subset that tries to avoid redundancy.

The prediction performance of the five algorithms on the test set is shown in Table 2. Our model produced the best performance with an AUC score of 0.773 on test set. In addition, the number of features after dimensionality reduction is the smallest. In contrast, the other four feature selection methods produced a relatively lower AUC score and more selected features.

**Table 2:** Performance of S-ISOMAP compared with other feature selection methods on the test set

Method	SEN	SPE	PRE	F1	ACC	MCC	AUC
SVM-RFE (19)	0.423	0.763	0.555	0.478	0.625	0.197	0.635
mRMR (30)	0.538	0.711	0.569	0.549	0.642	0.251	0.696
RF-SFS (17)	0.654	0.737	0.629	0.642	0.703	0.388	0.709
VSURF (10)	0.678	0.776	0.672	0.669	0.736	0.431	0.704
S-ISOMAP (3)	**0.707**	**0.819**	**0.721**	**0.713**	**0.768**	**0.508**	**0.773**

The highest value in each column is shown in bold. The numbers in parentheses represent the feature dimensions after dimensionality reduction

**Fig. 3:**
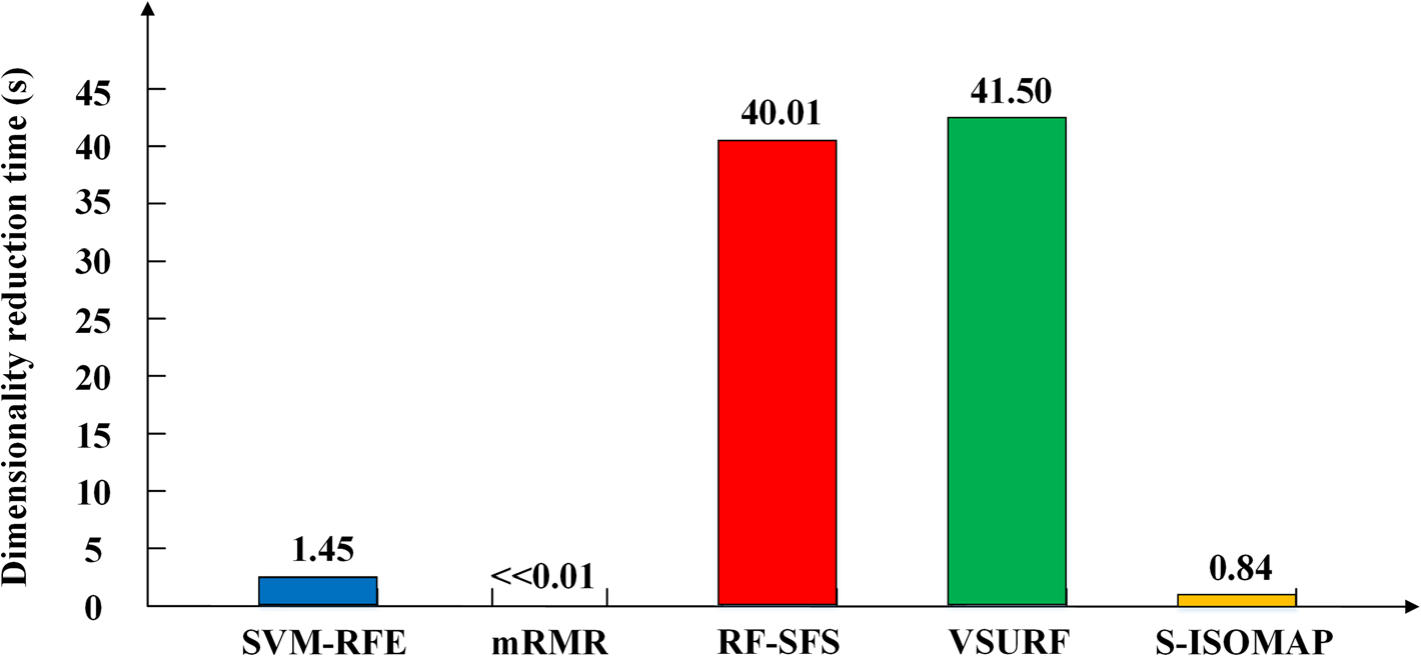
Running time of S-ISOMAP compared with other feature selection

Figure 3 shows the runtime comparison of S-ISOMAP with the other four feature selection methods. The dimensionality reduction time of mRMR is less than 0.01 (0.000001). The dimensionality reduction time of our method is only higher than that of mRMR, but lower than those of RF-SFS, SVM-RFE and VSURF.

### Compared with other methods

**Fig. 4:**
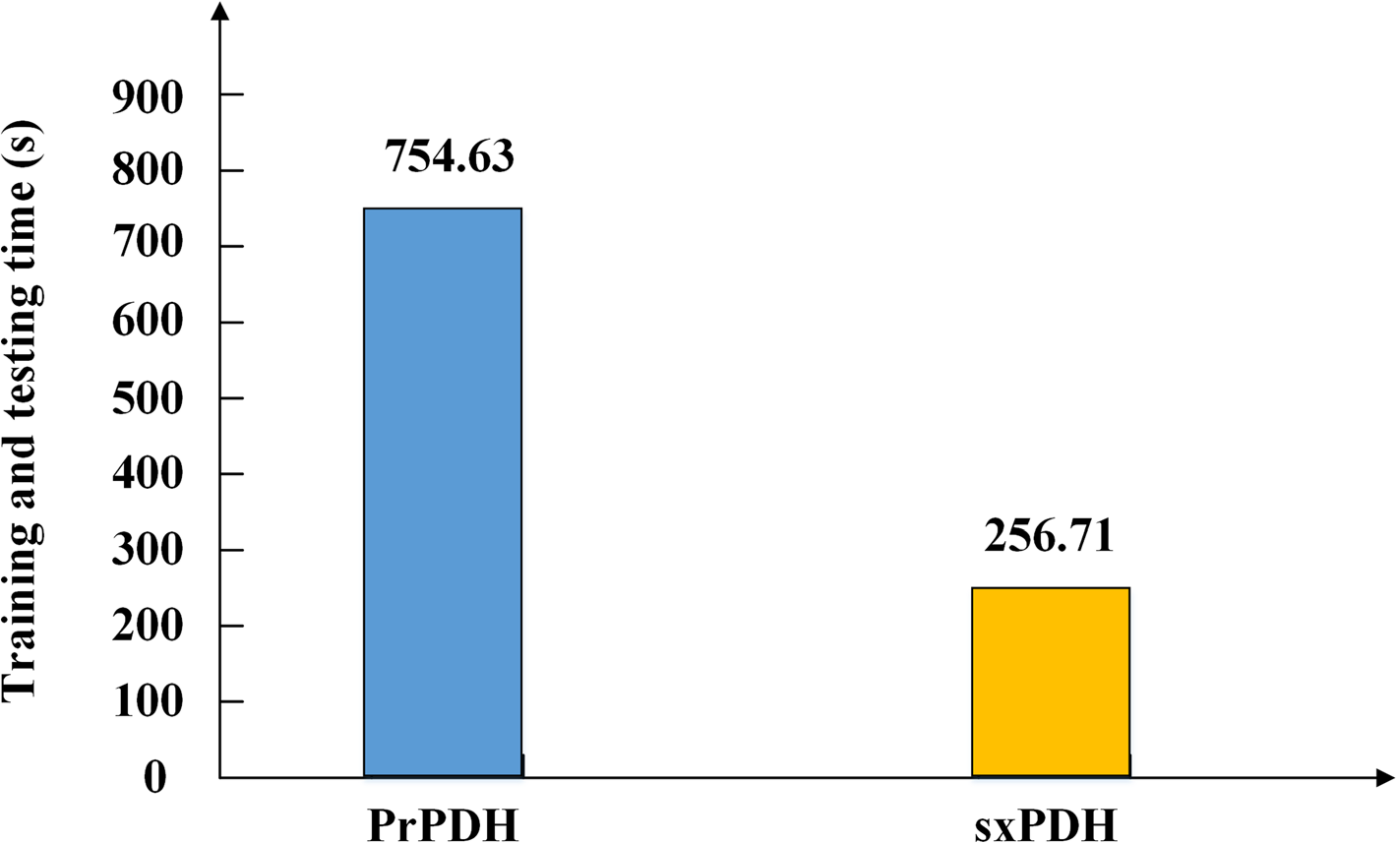
Running time of sxPDH compared with PrPDH

SAMPDI [29] and PremPDI [30] are two molecular mechanics-based approaches which can predict protein-DNA binding free energy changes, while mCSM-NA [31] uses the concept of graph-based signatures to quantitatively predict the influences of single mutation on protein-DNA or protein-RNA binding affinities. Recently, we proposed a computational methods called PrPDH [11] to predict DNA-binding hot spots, which uses VSURF method for feature selection and SVM as the classifier model. The comparison of our method sxPDH with these four methods is shown in Table 3. Our method sxPDH shows similar success rate in comparison with PrPDH. On the test set, the F1 score, MCC, ACC and AUC of our model sxPDH were 0.713, 0.508, 0.768 and 0.773 respectively, while PrPDH could correctly identify DNA-binding hot spots with F1 score = 0.706, MCC = 0.511, ACC = 0.766 and AUC = 0.764. Since the experiments of SAMPDI, PremPDI and mCSM-NA were performed on their webserver, we only compared the time performance of sxPDH and PrPDH. Our method sxPDH is far less than PrPDH in terms of optimal feature number (Table 3) and running time (Fig. 4). Overall, our method sxPDH exerts impressive predictive and time efficiency in detecting hot spots in protein–DNA interaction interfaces.

**Table 3:** Performance of different methods on the test set

Method	SEN	SPE	PRE	F1	ACC	MCC	AUC
SAMPDI	0.654	0.658	0.567	0.607	0.656	0.307	0.690
PremPDI	0.577	0.737	0.600	0.588	0.672	0.316	0.708
mCSM-NA	0.538	0.737	0.583	0.560	0.656	0.279	0.661
PrPDH	0.692	0.816	0.720	0.706	0.766	**0.511**	0.764
sxPDH	**0.707**	**0.819**	**0.721**	**0.713**	**0.768**	0.508	**0.773**

The highest value in each column is shown in bold

## Conclusion

In this work, we proposed a method called sxPDH based on S-ISOMAP and XGBoost to distinguish hot spots and non-hot spots at protein-DNA interfaces. Based on our previous work [11], 64 complexes were selected as the benchmark dataset, and 114 features were calculated from four types of feature groups. Then the feature dimension was reduced to three by S-ISOMAP method. The XGBoost was used to build the final prediction model. The prediction results show that the proposed method sxPDH has better prediction performance and lower time complexity. However, there is still room to improve our method. Because most used features in this study are related to proteins and amino acids, we will explore more DNA-related features to make our model more robust in the future work.

## Data Availability

The data and python code of sxPDH are freely available via GitHub: https://github.com/xialab-ahu/sxPDH.

## Abbreviations

S-ISOMAP: Supervised isometric feature mapping
XGBoost: Extreme gradient boosting
mRMR: Maximum relevance minimum redundancy
SFS: Sequential forward selection
ISOMAP: Isometric feature mapping
LLE: Local linear embedding
SLLE: Supervised locally linear embedding
RF-SFS: RF based on sequential forward selection
SVM-RFE: SVM-based recursive feature elimination
VSURF: Variable selection using random forests
SEN: Sensitivity
SPE: Specificity
PRE: Precision
F1: F1 score
ACC: Accuracy
MCC: Matthews correlation coefficient
AUC: The area under the ROC curve

## References

[1] Zhang J, Zhang Z, Chen Z, Deng L (2017). Integrating multiple heterogeneous networks for novel lncRNA-disease association inference. IEEE/ACM Trans Comput Biol Bioinform.

[2] König J, Zarnack K, Luscombe NM, Ule J (2012). Protein–RNA interactions: new genomic technologies and perspectives. Nat Rev Genet.

[3] Clackson T, Wells JA (1995). A hot spot of binding energy in a hormone-receptor interface. Science.

[4] Moreira IS, Fernandes PA, Ramos MJ (2007). Hot spots—a review of the protein–protein interface determinant amino-acid residues. Proteins.

[5] Xia J, Yue Z, Di Y, Zhu X, Zheng C-H (2016). Predicting hot spots in protein interfaces based on protrusion index, pseudo hydrophobicity and electron-ion interaction pseudopotential features. Oncotarget.

[6] Pan Y, Wang Z, Zhan W, Deng L (2017). Computational identification of binding energy hot spots in protein–RNA complexes using an ensemble approach. Bioinformatics.

[7] Qiao Y, Xiong Y, Gao H, Zhu X, Chen P (2018). Protein-protein interface hot spots prediction based on a hybrid feature selection strategy. BMC Bioinformatics.

[8] Deng L, Sui Y, Zhang J (2019). XGBPRH: prediction of binding hot spots at protein–RNA interfaces utilizing extreme gradient boosting. Genes.

[9] Wang L, Liu Z-P, Zhang X-S, Chen L (2012). Prediction of hot spots in protein interfaces using a random forest model with hybrid features. Protein Eng Des Sel.

[10] Xiong Y, Zhu X, Dai H, Wei DQ (2018). Survey of computational approaches for prediction of DNA-binding residues on protein surfaces. Methods Mol Biol.

[11] Zhang S, Zhao L, Zheng C-H, Xia J. A feature-based approach to predict hot spots in protein–DNA binding interfaces. Brief Bioinform. 2019. 10.1093/bib/bbz037. 10.1093/bib/bbz037 30957840

[12] Li J, Cheng K, Wang S, Morstatter F, Trevino RP, Tang J, Liu H (2018). Feature selection: a data perspective. ACM Comput Surv.

[13] Cai J, Luo J, Wang S, Yang S (2018). Feature selection in machine learning: a new perspective. Neurocomputing.

[14] Tenenbaum JB, De Silva V, Langford JC (2000). A global geometric framework for nonlinear dimensionality reduction. Science.

[15] Roweis ST, Saul LK (2000). Nonlinear dimensionality reduction by locally linear embedding. Science.

[16] Geng X, Zhan D-C, Zhou Z-H (2005). Supervised nonlinear dimensionality reduction for visualization and classification. IEEE Trans Syst Man Cybern B Cybern.

[17] Chen T, Guestrin C (2016). Xgboost: A scalable tree boosting system. Proceedings of the 22nd ACM Sigkdd International Conference on Knowledge Discovery and Data Mining.

[18] Borg I, Groenen P (2003). Modern multidimensional scaling: theory and applications. J Educ Meas.

[19] Chen Z, Liu X, Li F, et al. Large-scale comparative assessment of computational predictors for lysine post-translational modification sites. Brief Bioinform. 2018. 10.1093/bib/bby089. 10.1093/bib/bby089 PMC6954452 30285084

[20] Li F, Li C, Marquez-Lago TT (2018). Quokka: a comprehensive tool for rapid and accurate prediction of kinase family-specific phosphorylation sites in the human proteome. Bioinformatics.

[21] Li F, Wang Y, Li C, et al. Twenty years of bioinformatics research for protease-specific substrate and cleavage site prediction: a comprehensive revisit and benchmarking of existing methods. Brief Bioinform. 2018. 10.1093/bib/bby077. 10.1093/bib/bby077 PMC6954447 30184176

[22] Song J, Wang Y, Li F (2018). iProt-sub: a comprehensive package for accurately mapping and predicting protease-specific substrates and cleavage sites. Brief Bioinform.

[23] Song J, Li F, Leier A (2017). PROSPERous: high-throughput prediction of substrate cleavage sites for 90 proteases with improved accuracy. Bioinformatics.

[24] De Ridder D, Kouropteva O, Okun O, et al. Supervised locally linear embedding. In: Artificial Neural Networks and Neural Information Processing—ICANN/ICONIP: Springer; 2003. p. 333–41.

[25] Lou W, Wang X, Chen F, Chen Y, Jiang B, Zhang H (2014). Sequence based prediction of DNA-binding proteins based on hybrid feature selection using random forest and Gaussian naive Bayes. PLoS One.

[26] Peng H, Long F, Ding C (2005). Feature selection based on mutual information criteria of max-dependency, max-relevance, and min-redundancy. IEEE Trans Pattern Anal Mach Intell.

[27] Guyon I, Weston J, Barnhill S, Vapnik V (2002). Gene selection for cancer classification using support vector machines. Mach Learn.

[28] Genuer R, Poggi J-M, Tuleau-Malot C (2015). VSURF: an R package for variable selection using random forests.

[29] Peng Y, Sun L, Jia Z, Li L, Alexov E (2017). Predicting protein–DNA binding free energy change upon missense mutations using modified MM/PBSA approach: SAMPDI webserver. Bioinformatics.

[30] Zhang N, Chen Y, Zhao F (2018). PremPDI estimates and interprets the effects of missense mutations on protein–DNA interactions. PLoS Comput Biol.

[31] Pires DEV, Ascher DB (2017). mCSM-NA: predicting the effects of mutations on protein-nucleic acids interactions. Nucleic Acids Res.

